# *UGT440A1* Is Associated With Motility, Reproduction, and Pathogenicity of the Plant-Parasitic Nematode *Bursaphelenchus xylophilus*

**DOI:** 10.3389/fpls.2022.862594

**Published:** 2022-05-31

**Authors:** Min Wang, Guicai Du, Junna Fang, Linsong Wang, Qunqun Guo, Tingting Zhang, Ronggui Li

**Affiliations:** ^1^Medical College, Qingdao University, Qingdao, China; ^2^College of Life Sciences, Qingdao University, Qingdao, China; ^3^Qingdao JiMo People’s Hospital, Qingdao, China; ^4^Institute of Oceanology, Chinese Academy of Sciences, Qingdao, China

**Keywords:** *Bursaphelenchus xylophilus*, UDP-glycosyltransferase, motility, reproduction, pathogenicity

## Abstract

Pine wilt disease (PWD) caused by *Bursaphelenchus xylophilus* is considered a major threat to pine forests worldwide. Uridine diphosphate (UDP)-glycosyltransferases (UGTs) catalyze the conjugation of small lipophilic compounds with sugars and play crucial roles in the detoxification and homeostatic processes in all living organisms. We investigated the molecular characteristics and biological functions of the gene *UGT440A1* that encodes UGTs in *B. xylophilus*. The *in situ* hybridization results indicated that *UGT440A1* is expressed in all developmental stages of *B. xylophilus*, particularly in the head, intestine, and hypodermis of the second-stage of juveniles (J2), third-stage of juveniles (J3) and fourth-stage of juveniles (J4) females and in almost the whole body of J4 males and adults. Recombinant UGT440A1 was observed mainly in the inclusion bodies, and the enzyme activity assay revealed that UGT440A1 could catalyze the glycosylation reaction of two types of flavonols (kaempferol and quercetin). RNA interference (RNAi) of *UGT440A1* suppressed motility, feeding, and reproduction of *B. xylophilus*. Furthermore, *UGT440A1* knockdown caused a delay in the development of PWD symptoms in the pine seedlings inoculated with the nematodes. These results suggest that *UGT440A1* is involved in the pathogenic process of *B. xylophilus* and the information may facilitate a better understanding of the molecular mechanism of PWD.

## Introduction

Pine wilt disease (PWD) caused by pine wood nematode (PWN) *Bursaphelenchus xylophilus* is one of the most serious diseases that has caused considerable damage to pine forests in East Asia, North America, and Europe (Nickle et al., 1981; Mamiya, 1983; Mota et al., 1999; Penas et al., 2004; Zhao et al., 2008; Futai, 2013). Owing to the rapid spread of PWD and high mortality of host trees, this nematode is considered a major threat to pine forests worldwide and has been extensively studied (Futai, 2013; Modesto et al., 2021; Shinya et al., 2021). Despite several attempts to control the spread of the disease, no effective control method is available to date because of the poorly understood pathogenic mechanism of PWD (Jones et al., 2008). In Asia, *B. xylophilus* is transmitted from dead to healthy pine trees by the vector beetles. The nematode migrates to pine trees through wounds created by the feeding of insect vector, *Monochamus spp*. (Futai, 2013). The life cycle of the nematode, following invasion, comprises a phytophagous phase and a mycophagous phase. In the phytophagous phase, the nematode infects a tree, feeds on the epithelial cells, and leads to lethal wilting. Subsequently, the infected pines produce ethanol, monoterpenes and other volatile compounds (Ikeda and Oda, 1980). In our recent study, we found that a low concentration of ethanol derived by the host tree could promote the growth of *B. xylophilus* population and that a UGT gene-encoded enzyme UDP-glycosyltransferase (UGT) was significantly upregulated when PWNs were treated with ethanol (Wang et al., 2022).

UGTs are generally present in diverse organisms from bacteria to humans. These enzymes catalyze the transfer of glucose from UDP–glucose or other substrate donors to the substrate acceptors and play a crucial role in detoxification of endogenous and exogenous substances (Hundle et al., 1992; Bock, 2003). *B. xylophilus* must resist or metabolize the nematicidal substances produced by the pines once it invades a pine tree (Mamiya, 2012). The detoxification process mediated by these nematodes has been divided into three phases: the addition of functional groups to molecules (phase I); the actual detoxification reactions (phase II); and efflux (phase III) (Lindblom and Dodd, 2009). UGTs are one of the two main families of enzymes involved in phase II. Studies on UGTs in nematodes have mainly focused on investigating nematode resistance to xenobiotics. In parasitic nematodes, the increased activity of UGTs could protect the nematodes against drug toxicity and contribute to drug resistance (Vokral et al., 2012). Fontaine and Choe reported a resistance-related UGT that conferred high tolerance toward albendazole to *Caenorhabditis elegans* (Fontaine and Choe, 2018). Additionally, the investigation of the UGT family in *H. contortus* revealed significant sex differences in the expression levels of several UGTs (Matouskova et al., 2018). However, the molecular characteristics and biological functions of UGT gene in the nematodes remain poorly understood.

Based on the transcriptome analysis results for *B. xylophilus* related to the response to host-derived ethanol, we hypothesize that the upregulated UGT gene might not only participate in the detoxification process but also be associated with the reproduction of *B. xylophilus*. We cloned the gene according to the sequence obtained by the transcriptome analysis and submitted the sequences to the UDP-glycosyltransferase Nomenclature Committee^1^ to assign a name to the gene. The gene was named as *UGT440A1* by the committee. Furthermore, to elucidate the molecular and biological functions of *UGT440A1* in *B. xylophilus*, we expressed the recombinant UGT440A1, examined its spatiotemporal expression in different developmental stages of *B. xylophilus*, and investigated the influences on motility, reproduction, and pathogenicity of *B. xylophilus* after suppression of *UGT440A1* through RNA interference (RNAi).

## Materials and Methods

### Culture and Collection of Nematodes

The highly virulent *B. xylophilus* strain AMA3 was isolated from diseased *Pinus thunbergii* seedlings in Nanjing, China. The nematodes were cultured on *Botrytis cinerea* in potato dextrose agar (PDA) plates at 25°C in the dark for 7–9 days at Qingdao University (Qingdao, Shandong Province, China) (Guo et al., 2017). Then, the mixed-stage nematodes were separated using the Baermann funnel technique (Viglierchio and Schmitt, 1983). The nematodes were placed in Petri dishes for 1 h at 25°C in the dark to obtain eggs, as described previously (Futai, 1980; Tang et al., 2020). The collected eggs were hatched in the absence of food for 24 h in the dark at 25°C to obtain J2 larvae. Subsequently, the J2 larvae were cultured for 48 h at 25°C in the dark to obtain J4 worms. After the male and female J4 nematodes were distinguished, they were transferred to the PDA plates containing *B. cinerea* separately for 24 h at 25°C in the dark. Then, virgin adult worms were collected using two Baermann funnels (Zhu et al., 2016). Two-year-old *Pinus thunbergii* (*P. thunbergii*) seedlings were grown in the greenhouse at 25°C.

### *UGT440A1* Gene Cloning

DNA was extracted from nematodes with the method described previously (Huang et al., 2010). Total RNA was extracted using TRIzol^®^ Reagent (Invitrogen, Waltham, MA, United States) and treated with DNase I (Cwbio, Beijing, China), according to the manufacturer’s protocol. DNA and RNA were examined through electrophoresis on a 1.5% denaturing agarose gel and quantified by measuring ultraviolet absorbance at 260/280-nm wavelength (NanoDrop^®^ ND-2000, Thermo Fisher, USA). The synthesis of first-strand cDNA was performed, as described previously (Tang et al., 2020). According to the *UGT440A1* sequence obtained through the transcriptome analysis and the *B. xylophilus* genome data from WormBase ParaSite^2^, a pair of specific primers (forward: 5′-CCGGAATTCATTTAACCAACATAATATGTTG-3′; reverse: primer 5′-GCTCTAGAAAAGCTATTTATTAATTCTATTCAGA CA-3′) was used to amplify the complete *UGT440A1* through polymerase chain reaction (PCR) from *B. xylophilus* genomic DNA. The PCR conditions were as follows: pre-denaturation at 94°C for 5 min, followed by 30 cycles of denaturation at 94°C for 45 s; annealing at 56°C for 45 s; extension at 72°C for 150 s, and a final extension at 72°C for 10 min. To further analyze the UGT440A1 protein, the coding sequence of the gene was amplified by PCR using cDNA reverse-transcribed from mRNA of *B. xylophilus*. The primers (forward: 5′-CCCAAGCTTATGCGCGCCTTTCTGC −3′; reverse: 5′-CC GGAATTCTTACTCCGCTTTGACCTTGG-3′) were designed based on the ORF region of *UGT440A1*. The PCR conditions were as follows: pre-denaturation at 94°C for 5 min, followed by 30 cycles of denaturation at 94°C for 45 s; annealing at 60°C for 45 s; extension at 72°C for 90 s, and a final extension at 72°C for 10 min. The amplified products were cloned into a pMD^®^18-T Simple Vector (Takara, Dalian, China) and then sequenced.

### Sequence Analysis of *UGT440A1*

The program open reading frame (ORF) Finder^3^ was utilized to analyze the ORF of *UGT440A1*. The *B. xylophilus* genome from WormBase ParaSite was used for the structural analysis of *UGT440A1.* The protein sequences of *UGT440A1* were deduced from the nucleotide sequences by using the Basic Local Alignment Search Tool^4^. Protein sequences of other species utilized in alignments were obtained from the NCBI^5^. Conserved domains were identified using the conserved domain database of NCBI^6^. DNAMAN9 software was used to perform multiple alignments, and MEGA-X software was utilized to construct a phylogenetic tree with the neighbor joining method. Physicochemical characteristics of *UGT440A1* were obtained from ProtParam^7^. Signal peptides and transmembrane helices were predicted using SignalP Server^8^ and THHMM Server^9^, respectively. The deduced amino acid sequence of *UGT440A1* was modeled using the SWISS-MODEL program^10^ and further analyzed using the PyMOL 2.3.2 software.

### Fluorescence *in situ* Hybridization

mRNA FISH was performed to determine the spatial expression patterns of *UGT440A1* at different developmental stages of *B. xylophilus*. A red fluorescence-labeled probe (5′-Cy3-CGCTGATAGTAAGTCATCTCGCTTCCATGATCGTCGTCC ATGCCT-3′) was generated from the cloned sequence of *UGT440A1* and designed using Primer Premier 5.0 software (Primer, Canada). *B. xylophilus* at different developmental stages were collected and centrifuged in 1.5-mL centrifuge tubes. The nematodes were fixed with the RNAse-free paraformaldehyde solution (4%) at 5°C for 16 h and then pretreated at room temperature for 4 h. Smeared 50 μL suspension of nematodes (approximately 200 nematodes) in a microscope slide and baked the nematodes at 56°C on a slide warmer. FISH was performed using FISH *in situ* hybridization kit C007 (Gefan, Shanghai, China), according to the manufacturer’s protocol. The nematodes were incubated in 200 mM hydrochloric acid for 15 min in a humidified box containing 5 × saline sodium citrate (SSC) and formamide, and then washed twice with diethypyrocarbonate (DEPC)-treated water. Proteinase K (0.5 mg mL^–1^) was used to digest the nematodes at 37°C for 20 min, followed by washing with 100 mM glycine for 1 min and twice washing with phosphate buffer saline (PBS) for 2 min. The nematodes were incubated in RNAse-free paraformaldehyde solution (4%) for 10 min, and then washed twice with PBS for 3 min. Incubation of the nematodes with acetic anhydride (0.25%) for 10 min were performed to reduce the non-specific binding, following five times washing with PBS for 5 min and twice washing with 5 × SSC for 2 min. The nematodes then were pre-hybridized in hybridization buffer at 65°C for 1 h. *In situ* hybridization was performed with rotation and probe concentration of 1.0 μg mL^–1^ for 48 h at 65°C. After hybridization, the nematodes were washed three times with a mixture of formamide and 4 × SSC (1:1) at 65°C for 15 min and five times with PBS for 5 min at room temperature. Counterstaining was carried out with 4′,6-diamidino-2-phenylindole (2.0 μg mL^–1)^ for 5 min. The nematodes were examined under a Nikon light microscope (Eclipse Ci, Nikon, Japan). The sense probe (5′-Cy3-AGGCATGGACGACGATCATGGAAGCGAGATGACTTACTA TCAGCG-3′) was used as a negative control.

### Expression and Purification of UGT440A1

The *UGT440A1* coding region without the putative signal sequence was amplified through PCR from the original plasmid by using specific primers (forward: 5′- ATGGA GAAGATCTTACTCTTAAATGCGGCGAGAATC-3′; reverse: 5′- ATTGCCGACGCCGTGATTACTT-3′) and inserted into the pET-15b (Invitrogen, United States) vector. Plasmid was transformed into *Escherichia coli* (*E. coli*) BL21 (DE3) Plyss (Solarbio, Beijing, China). Expression of the recombinant UGT and collection of inclusion bodies were performed using the method described in a study, with some modifications (Liu et al., 2015). The recombinant UGT440A1 was overexpressed in *E. coli* BL21 (DE3) harboring pET-15b-UGT through Isopropyl-β-D-thiogalactopyranoside (IPTG) induction at 37°C. The inclusion bodies were dissolved in 10 mL solution buffer (20 mM Tris, 5 mM DTT, 8 M urea, pH 8.0) at 4°C overnight. The supernatant was collected through centrifugation at 10000 g for 15 min at room temperature and diluted with refolding buffer (20 mM Tris-HCl, 150 mM NaCl, pH 8.0) to gradually reduce the concentration of urea from 8 M to 1 M. The refolded protein was purified through Ni-NTA affinity chromatography and analyzed through SDS-PAGE (12% gel) (Liu et al., 2015).

### Activity Assay of UGT440A1 Recombinant Protein

UGT activity was assayed according to a method reported previously, with some modifications (Real et al., 1991). The reaction mixture, with the final volume of 0.2 mL, comprised 100 mM Tris-HCl buffer (pH 7.4), 10 mM MgCl_2_, 5 mM D-gluconic acid lactone, 500 μM uridine diphosphate-α-D-glucose (UDPG), 100 mM substrate (kaempferol or quercetin), and 100 μL recombinant UGT. The reaction mixtures were incubated for 1 h at 37°C, and the reaction was terminated by adding 1 vol. methanol. Proteins and insoluble materials were removed through centrifugation at 15,000 g for 5 min at 4°C. Ten microliter of the filtered supernatant fraction was used for high-performance liquid chromatography (HPLC) analysis of the reaction products. Reactions performed in the absence of UDPG or recombinant UGT440A1 served as the control.

### Double-Stranded RNA Synthesis

By using pET-15b-*UGT440A1* as the template, a 368-bp DNA fragment was amplified through PCR with T_7_-labeled gene-specific primers (forward: 5′-TAATACGACTCACTATAGGGAAGTAATCACGGCGTCGGC-3′; reverse: 5′-TAATACGACTCACTATAGGGAAGCACATGGT CTCTTCC-3′). A pair of T_7_-labeled gene-specific primers was used to amplify the 323-bp DNA fragment of green fluorescent protein gene (*Bxy*-*gfp*), with pET-15b-*gfp* as the template (forward 5′-TAATACGACTCACTATAGGGAACGG CCACAAGTTCAGC-3′; reverse 3′- TAATACGACTCACTATA GGGAAGTCGATGCCCTTCAGC-3′). The amplified products were used as the templates for double-stranded RNA (dsRNA) synthesis. The dsRNA was synthesized using the MEGAscript (TM) RNAi Kit (Invitrogen, Vilnius, Lithuania), according to the manufacturer’s instructions. Fluorescent dsRNA labeled with cyanine dyes 3 (Cy3) was constructed by adding Cy3-dCTP in the reaction system. The integrity of dsRNAs was visualized in a 1.5% agarose gel.

### Efficiency Assessment of RNAi

Approximately 4000 *B. xylophilus* (a mixture of juveniles and adults) were soaked in 50 μL soaking buffer (0.05% gelatin, 3 mM spermidine, 0.25 × Mg^2+^-free M9) containing *UGT* dsRNA (1.0 μg/μL) and then incubated for 48 h at 25°C (Huang et al., 2019; Park et al., 2008). Afterward, *B. xylophilus* were washed with sterile water several times. Nematodes soaked in soaking buffer containing ddH_2_O and *gfp* dsRNA were used as double negative controls. The nematodes were rinsed four times with sterilized water and photographed under a fluorescence microscope to assess the efficiency of the dsRNA uptake. QRT-PCR was used to verify the effect of *UGT440A1* silencing through RNAi on mRNA levels. PCR was performed using TB Green Premix Ex Taq II (TaKaRa, Dalian, China), with 0.4 μM each of forward and reverse primer (forward: 5′-CTCGCGGAAGCGGGTTACAA-3′; reverse: 5′-CTGGTCGCGACACCCAAGTT-3′). The actin gene amplified by the primer pair (forward: 5′-CTGCTGAGCGTGAAATCGT-3′ and reverse: 5′-GTTGTAGGTGGTCTCGTGGA-3′) was used as the internal control. The PCR program was set as follows: 30 s at 95°C, followed by 40 cycles of 5 s at 95°C and 34 s at 60°C. Each experiment was performed thrice.

### Assay for Motility After RNAi

Approximately 40 nematodes (a mix of juvenile and adult nematodes) were observed under a stereo microscope after the nematodes were soaked in buffer with dsRNA (as described above) for 48 h at 25°C in the dark. The head thrashing frequency (> 120° within a single head swing) in 30 s was used to measure the vitality of *B. xylophilus* (Tang et al., 2020). Nematodes soaked in buffer containing ddH_2_O and *gfp* dsRNA were used as double negative controls. Each assay was repeated three times.

### Assay for Feeding and Reproduction After RNAi

Twenty pairs of virgin male and female nematodes were soaked separately in buffer containing dsRNA (1.0 μg/μL) for 48 h (Huang et al., 2019). Thereafter, the nematodes were washed several times to remove external dsRNA. Then, 10 pairs of nematodes were cultured in a PDA plate with *B. cinerea* at 25°C in the dark. The feeding area of *B. xylophilus* was photographed daily. After culturing for 9 days, the nematodes were isolated from the PDA plates and counted under an optical microscope. The other 10 pairs were transferred onto a glass dish with ddH_2_O for 48 h in the dark at 25°C to allow the nematodes to lay eggs, and the number of eggs was counted under an optical microscope. To determine the effect of *UGT* dsRNA on egg hatching of *B. xylophilus*, thirty pairs of virgin male and female nematodes were soaked separately in buffer containing dsRNA for 48 h. Following treatment, the nematodes were transferred onto a glass dish for 16 h to lay eggs, then eggs were incubated at 25°C for 20 h for hatching, and the hatching rate was calculated (Huang et al., 2019). The same quantity of nematodes soaked in buffer containing ddH_2_O and *gfp* dsRNA served as negative controls. Each experiment was performed three times.

### Assay on the Pathogenicity After RNAi

Approximately 3000 *B. xylophilus* (a mix of juvenile and adult nematodes) were soaked in 50 μL soaking buffer containing *UGT* dsRNA (1.0 μg/μL) and then incubated for 48 h at 25°C (Huang et al., 2019). Afterward, *B. xylophilus* were washed with sterile water several times. Thereafter, the nematodes were inoculated into 2-year-old *P. thunbergii* seedlings. Sterile water without nematodes and nematodes soaked in buffer containing ddH_2_O and *gfp* dsRNA were used as negative controls. Each treatment was replicated three times. Each *P. thunbergii* seedling was inoculated with 200 μL suspension of mixed-stage nematodes (approximately 1,000 nematodes). Approximately 2-cm-long wounds on *P. thunbergii* at 30–50 cm above the soil level were inoculated and sealed with parafilm (Qiu et al., 2016). The inoculated *P. thunbergii* seedlings were grown in the greenhouse at 25°C. Wilting symptoms developing on the pine woods that were inoculated with the nematodes were observed daily. The PWD symptoms were evaluated and categorized into four groups: 0 = all of the needles were green; 1 = 0%–25% of the needles were discolored and turning yellow; 2 = 26%–50% of the needles had turned yellow; 3 = 51%–75% of the needles had turned yellow; and 4 = 76%–100% of the needles had turned yellow (Yu et al., 2012). The infection rates and the disease severity index (DSI) of the *P. thunbergii* seedlings were calculated with a method described previously (Yu et al., 2012).

### Statistical Analysis

All experiments involving *B. xylophilus* included three replicates, with each treatment replicated three times. All values of the repeated experiments are expressed as means ± standard deviation (S.D.), and all statistical analyses were performed using SPSS 19.0 software (SPSS, Chicago, IL, USA). The independent sample *t*-test and one-way analysis of variance were used to assess differences between the groups. A *P* value of < 0.05 was considered to denote statistical significance.

## Results

### Alignment and Phylogenetic Analysis of *UGT440A1*

**FIGURE 1:**
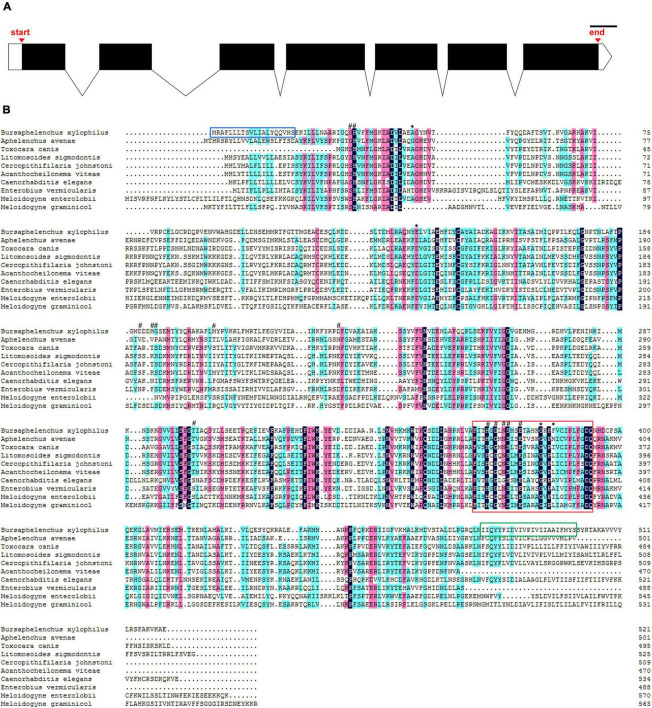
Sequence analysis of *UGT440A1*. **(A)** Genomic DNA structure of *UGT440A1*. White boxes indicate exons, the black region corresponds to the coding sequences, and the lines indicate introns; scale bar = 100 bp. **(B)** Alignment of *UGT440A1* with homologs. The alignment compares UGT440A1 (MZ467299) with the UGT proteins of *Aphelenchus avenae* (KAH7725749.1), *Toxocara canis* (KHN83392.1), *Litomosoides sigmodontis* (VDK69150.1), *Cercopithifilaria johnstoni* (CAG9530659), *Acanthocheilonema viteae* (VBB30777.1), *Caenorhabditis elegans* (NP_506211.1), *Enterobius vermicularis* (VDD95930.1), *Meloidogyne enterolobii* (CAD2191161.1) and *Meloidogyne graminicola* (KAF7639096.1). Black, red, and blue shadings indicate fully conserved, strongly conserved, and weakly conserved amino acids, respectively. Signal peptide sequences, transmembrane region, and typical signature of UGT are enclosed by the blue, green, and red boxes, respectively. The predicted active sites and polypeptide binding sites are indicated by (#) and (*), respectively.

**FIGURE 2:**
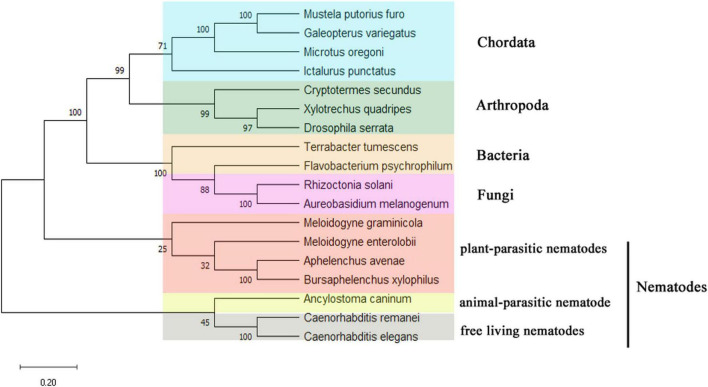
Unrooted phylogenetic analysis of UGT440A1 protein. Percentage nodal support is based on 500 bootstrap replicates. The scale bar indicates the evolutionary distance (0.2 substitutions per position). *Mustela putorius furo* (XP_004738075.1); *Galeopterus variegatus* (XP_008590698.1); *Microtus oregoni* (XP_041487187.1); *Ictalurus punctatus* (ABI51987.1); *Cryptotermes secundus* (XP_023705394.1); *Xylotrechus quadripes* (QIK00375.1); *Drosophila serrata* (XP_020813000.1); *Terrabacter tumescens* (GGM90420.1); *Flavobacterium psychrophilum* (WP_227038871.1); *Rhizoctonia solani* (CUA68035.1); *Aureobasidium melanogenum* (AQQ13387.1); *Meloidogyne graminicola* (KAF7639096.1); *Meloidogyne enterolobii* (CAD2191161.1); *Aphelenchus avenae* (KAH7725749.1); *Bursaphelenchus xylophilus* (MZ467299); *Ancylostoma caninum* (RCN52832.1); *Caenorhabditis remane* (KAF1754268.1); *Caenorhabditis elegans* (NP_506211.1).

The DNA sequence of *UGT440A1* was successfully amplified by PCR (Supplementary Figure 1). The sequence analysis showed that the PCR product comprises 2,231 bp containing a 1,566-bp ORF which encoded 521 amino acids. The structural analysis showed that *UGT440A1* (*BXY_1088500.1*) comprised seven exons and six introns and contained 50 bp of the 5′ untranslated region and 50 bp of the 3′ untranslated region (Figure 1A). The coding sequence of *UGT440A1* was submitted to NCBI Genbank under the accession number MZ467299. The conserved domain search showed that *UGT440A1* belonged to the glycosyltransferases B (GTB) superfamily (the members of this family share a common GTB topology) of glycosyltransferase and the coding sequence of *UGT440A1* consisted of the GT1-Gtf-like Pfam domain, which was localized between amino acids 22 and 443. The alignment of multiple amino acid sequences revealed the homology of UGT440A1 with a range of UGTs (Figure 1B). Furthermore, sequence analysis revealed the presence of a typical UGT signature sequence in *UGT440A1:* [FVA]-[LIVMF]-[TS]-[HQ]-[SGAC]-G-x(2)-[STG]-x(2)-[DE]-x(6)-P-[LIVMFA]-[LIVMFA]-x(2)-P-[LMVFIQ]-x(2)-[DE]-Q (the amino acids in the square brackets can be arbitrary, and x stands for any base) (Mackenzie et al., 1997; Figure 1B). The further bioinformatics analysis showed the presence of a signal peptide and a transmembrane region in the deduced amino acid sequence of *UGT440A1* (Figure 1B), which indicated that UGT440A1 is a transmembrane protein. The phylogenetic relationship of UGT440A1 with other UGT proteins revealed that UGT440A1 formed a well-founded cluster with UGT of other nematodes (Figure 2). And UGT proteins in nematode species apparently separated those of plant-parasitic nematodes, including *B. xylophilus*, *Aphelenchus avenae*, *Meloidogyne enterolobii* and *Meloidogyne graminicola* from those of other nematode species. Notably, the UGT protein of *Aphelenchus avenae* was the closest protein to UGT440A1 among the 18 family members examined. In addition, four outgroups, namely Chordata, Arthropoda, Bacteria, and Fungi were observed (Figure 2). The analysis showed that the UGT440A1 sequences are more closely related to fungal and bacterial sequences compared with animal sequences.

### Molecular Modeling of *UGT440A1*

**FIGURE 3:**
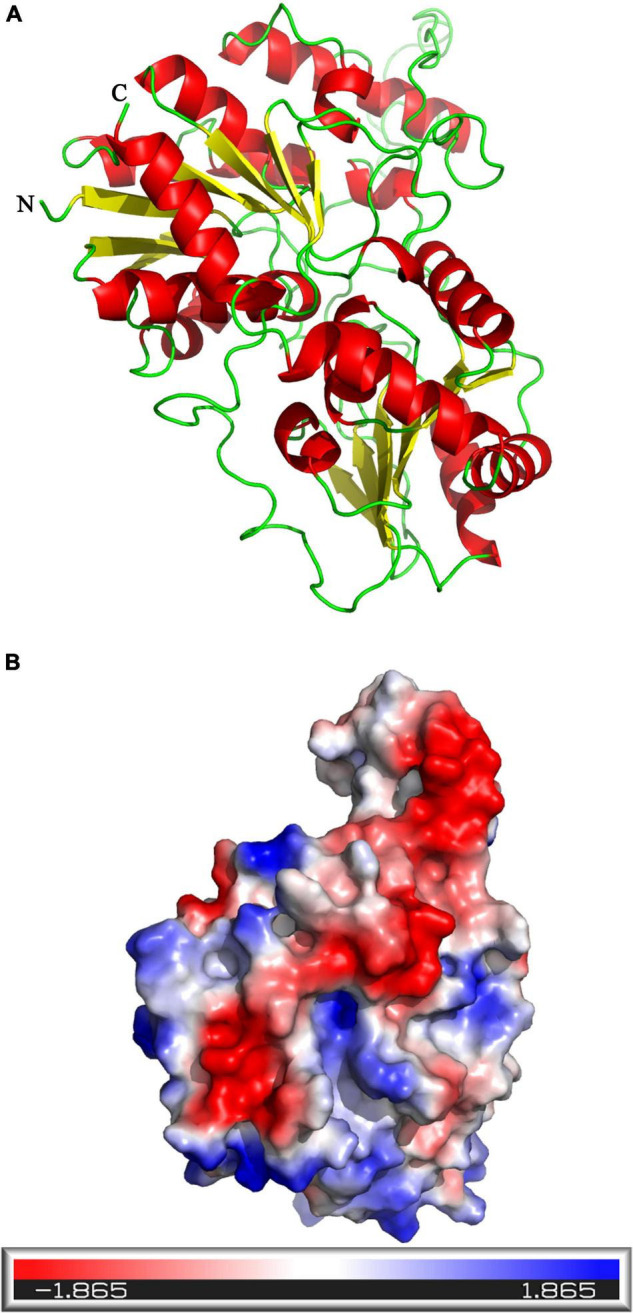
Predicted structure of *UGT440A1*. **(A)** Three-dimensional structure of *UGT440A1*, with macrolide glycosyltransferase (PDB ID: 2iya.1) as a template. **(B)** The electrostatic potential surface of *UGT440A1*. The color gradient ranging from red to blue corresponds to negatively charged regions (−5*K_*B*_T/e*) to positively charged regions (+ 5 *K_*B*_T/e*), and white color indicates neutral regions.

The tertiary structure of *UGT440A1* was predicted with the crystal structure of macrolide glycosyltransferase (PDB ID: 2iya.1) as a template by using the online program SWISS-MODEL (Figure 3A). The theoretical tertiary structure consisted of 15 alpha-helices and 13 beta-strands. The N-terminal domain had 7 beta-strands and surrounded by 8 alpha-helices, and the C-terminal domain was made up of 7 alpha-helices and 6 beta-strands. The molecular surface of *UGT440A1* was assessed using the PyMOL program, as the protein was in a cell environment. Molecular surface modeling revealed mainly the presence of white patches, along with slight red and blue patches, which indicated the presence of both neutral and charged regions (Figure 3B).

### Gene Expression Pattern of *UGT440A1* in PWNs

**FIGURE 4:**
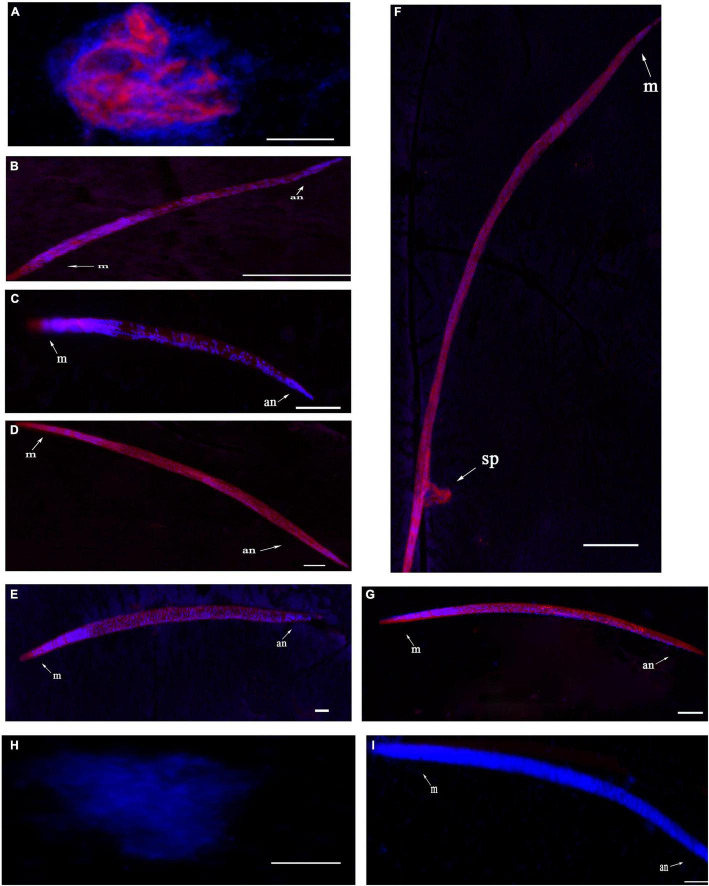
Localization of *UGT440A1* mRNA in *B. xylophilus* determined through fluorescence *in situ* hybridization. Hybridization with the red fluorescence-labeled probes in the embryo **(A)**, J2 juvenile **(B)**, J3 juvenile **(C)**, male J4 juvenile **(D)**, female J4 juvenile **(E)**, male adult **(F)**, and female adult **(G)**. Hybridization in embryo **(H)** and female adult **(I)** in the negative control. m, metacorpus; an, anus; sp, spicules. Scale bar = 20 μm **(A–E, H)**, 50 μm **(F,G,I)**.

Fluorescence *in situ* hybridization (FISH) was used to identify the spatiotemporal expression of *UGT440A1*. Hybridization signals were detected from embryo until adult *B. xylophilus* were formed. The red fluorescence-labeled probe stained nearly the whole embryo (Figure 4A), whereas a restricted staining pattern was observed in juvenile nematodes (J2, J3, and J4 females), wherein the hybridization signals were detected in the head, intestine, and subcutaneous tissues (Figures 4B,C,E). However, the hybridization signals were detected nearly throughout the body of J4 males and adults (Figures 4D,F,G). No hybridization signal was observed in the negative controls (Figures 4H,I).

### Expression, Purification, and Activity of the Recombinant *UGT440A1*

**FIGURE 5:**
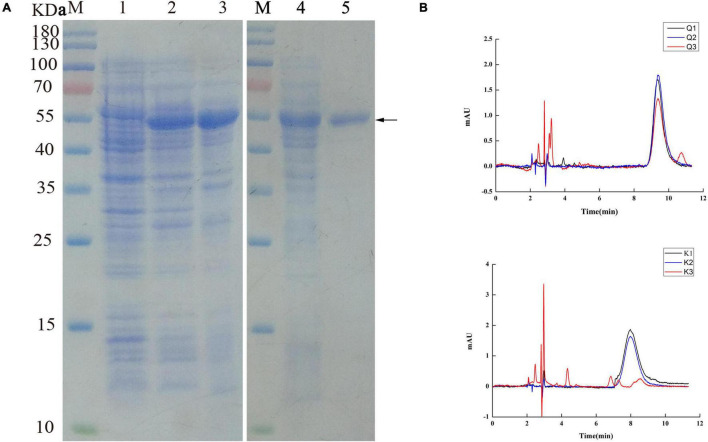
Purification and activity assay of the recombinant UGT440A1. **(A)** SDS-PAGE analysis of the expression and purification of recombinant UGT440A1. M, standard proteins; 1, total proteins of *E. coli* BL21 (DE3); 2, total proteins of *E. coli* BL21 (DE3) harboring pET-15b- *UGT440A1*; 3, inclusion bodies; 4, washed inclusion bodies;5, purified recombinant UGT440A1. **(B)** Detection of the recombinant *UGT440A1* activity through HPLC analysis. Overlapping chromatogram of reaction products, with quercetin (Q) or kaempferol (K) as the substrate; reaction performed in the absence of UDPG (Q1 and K1); the absence of recombinant UGT440A1 (Q2 and K2); and complete reaction mixtures (Q3 and K3).

The *UGT440A1* coding region without the putative signal sequence was amplified through PCR and cloned into the vector pET-15b to construct pET-15b-UGT. The recombinant UGT440A1 was overexpressed in *E. coli* BL21 (DE3) through IPTG induction at 37°C. The SDS-PAGE analysis indicated that the recombinant protein had the expected molecular mass of 53 kDa, which corresponded to 51.5 kDa from the *UGT440A1* ORF and 1.5 kDa encoded by the expression vector, including a His_6_tag (Figure 5A). The expressed recombinant protein was found mainly in the inclusion bodies, which could be refolded by stepwise dilution with refolding buffer (Figure 5A). The renatured recombinant UGT440A1 was then purified through Ni2 + affinity chromatography, and the electrophoretic homogeneity was verified through SDS-PAGE (Figure 5A). To assay the activity of UGT440A1, two types of flavonols, quercetin and kaempferol, which are the parent nuclei of flavonoids in pine wood, were selected as substrates to detect whether recombinant UGT440A1 had the catalytic activity. Because the polarity of flavanoids increases when they combine with glucose, the product is expected to peak earlier in case of a glycosylation reaction. The HPLC results indicated that the expressed recombinant protein could catalyze the two substrates, as more than one products were detected (Figure 5B).

### Efficiency Assessment of RNAi

**FIGURE 6:**
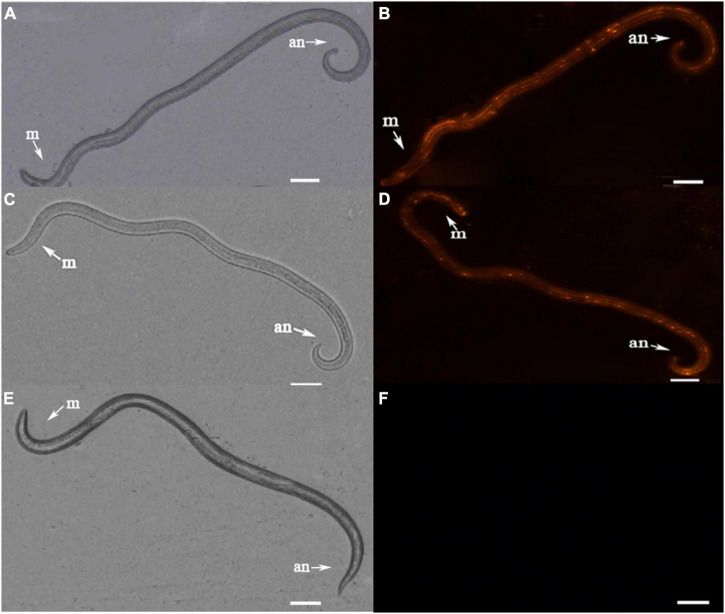
Fluorescence micrographs of *B. xylophilus* after RNAi. **(A)** Morphology of nematode soaked in *UGT440A1* dsRNA. **(B)** The red fluorescence indicates the Cy3-labeled *UGT440A1* dsRNA entry into the nematode. **(C)** Morphology of nematode soaked in *gfp* dsRNA. **(D)** The red fluorescence indicates the Cy3-labeled *gfp* dsRNA entry into the nematode. **(E)** Morphology of nematode soaked in sterilized water. **(F)** No fluorescence was detected in the nematode soaked in sterilized water. m, metacorpus; an, anus. Scale bar = 50 μm.

**FIGURE 7:**
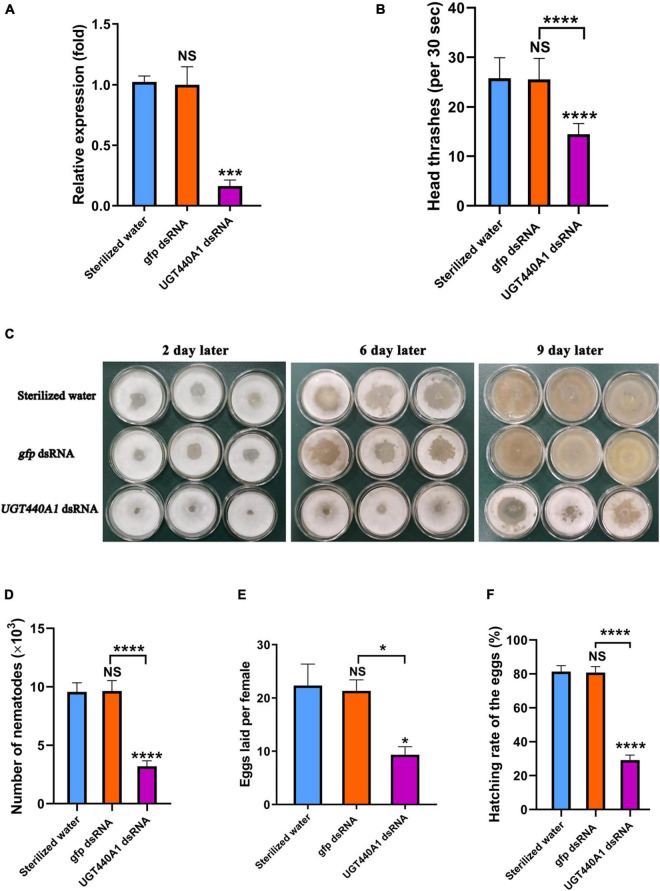
Expression and function analysis of *UGT440A1* after RNAi. **(A)** Relative expression of *UGT440A1* after RNAi. **(B)** The motility of *B. xylophilus* assessed on the basis of the number of head thrashes per 30 s after RNAi. **(C)** Feeding of *B. cinerea* by *B. xylophilus* soaked in sterilized water, *gfp* dsRNA, and *UGT440A1* dsRNA solutions. **(D)** Reproductive assay of *B. xylophilus* after RNAi. **(E)** The ability of egg laying measured by the number of eggs laid per female after RNAi. **(F)** The ability of egg hatching after RNAi. Asterisks indicate statistically significant differences (*P < 0.05, ***P < 0.001 and ****P < 0.0001), and NS denotes no significant difference.

To verify that the dsRNA could be transferred effectively into *B. xylophilus*, nematodes were observed under a fluorescence microscope. The patterns of fluorescein indicated that Cy3 labeled dsRNA was taken up by the nematodes effectively (Figures 6B,D). No fluorescence was detected in the nematode soaked in sterilized water (Figure 6F). And no phenotypic changes were detected in the nematodes after RNAi of *UGT440A1* (Figures 6A,C,E). The RNAi efficiency on the *UGT440A1* expression level was evaluated through QRT-PCR. The results showed that compared with the expression level of *UGT440A1* in the nematodes soaked in sterilized water, that in the nematodes soaked in *UGT440A1* dsRNA was only 0.15 (Figure 7A) (*p* < 0.001). This result indicated that the expression of *UGT440A1* in nematodes could be strongly inhibited by soaking the nematodes in *UGT440A1* dsRNA. The *gfp* dsRNA, as one of the negative controls, demonstrated no significant effects on the expression level of *UGT440A1* compared with sterilized water (Figure 7A) (*p* > 0.05).

### *UGT440A1* Is Required for Motility of *Bursaphelenchus xylophilus*

To analyze the influence of *UGT440A1* on the locomotion of *B. xylophilus*, the head thrashing frequency of the nematodes was observed under a stereo microscope. The nematodes soaked in dsRNA exhibited difficulties in motility. The head thrashing frequencies in the *UGT440A1* dsRNA, sterilized water, and *gfp* dsRNA groups were 14.53 ± 0.19, 25.82 ± 0.37, and 25.59 ± 0.38 times per 30 s, respectively (Figure 7B). These results indicated that *UGT440A1* is crucial for *B. xylophilus* motility.

### *UGT440A1* Is Crucial for Feeding and Reproduction of *Bursaphelenchus xylophilus*

The nematode feeding areas were observed after the nematodes soaked in the dsRNA of *UGT440A1* were transferred to the PDA plate. The feeding rate of the treatment groups was obviously slower than that of the negative controls (Figure 7C). At the ninth day, almost all of the hyphae had been consumed by *B. xylophilus* in the sterilized water and *gfp* dsRNA groups, whereas in the *UGT440A1* dsRNA group, only a part of the hyphae was consumed (Figure 7C). The same day, the nematodes were collected and counted in each group; the number of nematodes in the *UGT440A1* dsRNA, sterilized water and *gfp* dsRNA groups was 3,192 ± 111, 9,572 ± 182, and 9,642 ± 210, respectively (Figure 7D). Sterilized water and *gfp* dsRNA were not found to have significant influences on the feeding and reproduction of *B. xylophilus* (*p* > 0.05). These results indicated that *UGT440A1* is essential for feeding and reproduction of *B. xylophilus*.

Furthermore, to evaluate the effects of *UGT440A1* on egg laying and hatching abilities of the nematodes, we treated the virgin adults with *UGT440A1* dsRNA. The number of eggs laid by an individual female nematode in the treatment, sterilized water, and *gfp* dsRNA groups was 9.33 ± 1.52, 22.33 ± 4.04, and 21.33 ± 2.08, respectively (Figure 7E). The hatching rates for the *UGT440A1* dsRNA, sterilized water, and *gfp* dsRNA groups were 29.11% ± 3.01%, 81.33% ± 3.53%, and 80.78% ± 3.49%, respectively (Figure 7F). These results suggested that *UGT440A1* is vital for the egg laying and hatching potential of *B. xylophilus*.

### *UGT440A1* Is Involved in *Bursaphelenchus xylophilus* Pathogenicity

**FIGURE 8:**
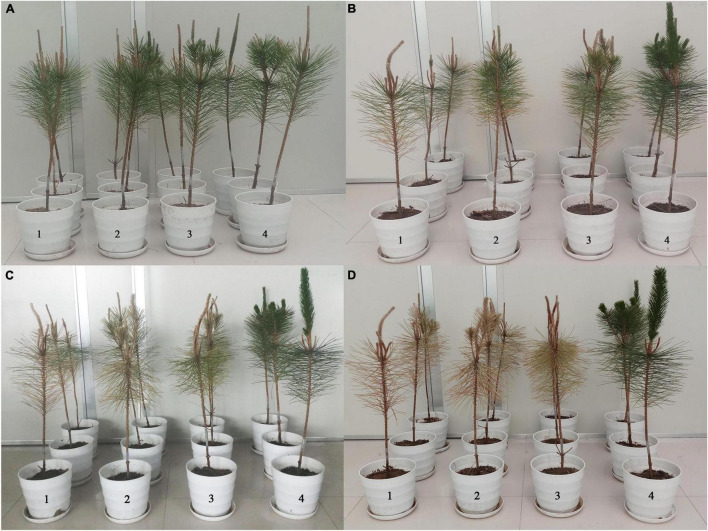
Pathogenicity assay of *B. xylophilus* after RNAi of *UGT440A1*. Wilting symptoms in *P. thunbergii* seedlings produced at 1 day **(A)**, 20 days **(B)**, 40 days **(C)**, and 60 days **(D)** after inoculation with *B. xylophilus* soaked in sterilized water (1), *gfp* dsRNA (2), *UGT440A1* dsRNA (3), and sterilized water without *B. xylophilus* (4).

To examine the virulence of the nematodes, *B. xylophilus* treated with *UGT440A1* dsRNA were inoculated into 2-year-old *P. thunbergii* seedlings (Figure 8A). Twelve days after inoculation, the pine seedlings inoculated with nematodes that were treated with sterile water and *gfp* dsRNA exhibited clear symptoms of PWD. The infection rate of both the sterilized water and *gfp* dsRNA treatment groups was 33.3%, whereas the DSI for the two groups was 16.67 ± 16.67 and 8.33 ± 8.33, respectively (Table 1) (*p* < 0.05). The *UGT440A1* dsRNA treatment group exhibited no PWD symptom in at this time point; however, this group exhibited leaf yellowing 18 days after inoculation. Furthermore, the pine seedlings inoculated with nematodes that were treated with sterilized water and *gfp* dsRNA exhibited higher infection rates and DSI, respectively, than those treated with *UGT440A1* dsRNA at 20 and 40 days after inoculation (Table 1 and Figures 8B,C) (*p* < 0.05). On day 60, all the pine seedlings in the sterilized water and *gfp* dsRNA groups died, whereas the *UGT440A1* dsRNA-treated seedlings were still alive, despite the obvious symptoms of PWD (Figure 8D). The pine seedlings inoculated with sterile water without nematodes did not exhibit any PWD symptom throughout the experimental period. These results indicated that PWD onset was delayed after RNAi of *UGT440A1*.

**TABLE 1:** Infection rates and disease severity index of *Pinus thunbergii* seedlings inoculated with *Bursaphelenchus xylophilus* under different treatments.

Treatment	Infection Rates (%)				Disease Severity Index (DSI)			
	12th Day	20th Day	40th Day	60th Day	12th Day	20th Day	40th Day	60th Day
**ddH_2_O**	33.3	66.6	100	100	16.67 ± 16.67^a^	41.67 ± 9.64^a^	68.81 ± 3.52^a^	100 ± 0.00^a^
**gfp dsRNA**	33.3	66.6	100	100	8.33 ± 8.33^a^	27.78 ± 8.98^a^	62.43 ± 4.91^a^	100 ± 0.00^a^
***UGT440A1*** **dsRNA**	0	33.3	66.6	100	0 ± 0.00^a^	13.89 ± 6.33*^b^*	37.50 ± 6.87^b^	100 ± 0.00^a^

*Different letters indicate significant differences (p < 0.05) among treatments.*

## Discussion

UGTs catalyze the conjugation of small lipophilic compounds with sugars and play vital roles in the detoxification and homeostatic processes in all living organisms (Meech et al., 2012; Guillemette, 2003). The nucleotide sugars could be UDP–galactose, UDP–glucose, UDP–glucolonic acid, UDP–xylose, or UDP–rhamnose (Mackenzie et al., 1997). Mammalian UGTs mainly use UDP–glucuronic acid as the sugar donor, whereas invertebrate and plant UGTs mainly use glucose. Although UGTs in plants and mammals, particularly in humans, have been studied extensively in the past decades, little is known about the molecular features and biological functions of UGTs in *B. xylophilus* (Bock, 2016). In this study, we reported a *UGT440A1* gene encoding UGT from *B. xylophilus* and investigated the molecular features and biological roles of this gene for the first time.

UGT440A1 is evolutionarily conserved across species of different phyla. The relatively conserved functional domain of *UGT440A1* protein was found in the region of the *UGT440A1* signature motif, which was located at the C terminal. Multiple alignments of the UGT protein sequences revealed that the C-terminal domain (sugar donor-binding domain) was more conserved than the N-terminal domain (substrate-binding domain), which might be related to the diversity of substrate structures. The phylogenetic analysis showed that UGT440A1 forms a branch with UGT of other nematodes, which revealed a nematode-specific divergence for UGTs. UGT440A1 and its homolog UGTs from *Aphelenchus avenae*, *Meloidogyne enterolobii* and *Meloidogyne graminicola* were grouped in the same sub-clade, which was separated from all the other nematode homologs. UGT440A1 is closely related to the UGT gene of the Aphelenchus avenae. A recent study reported that compared to the other nematode species, *B. xylophilus* and *Aphelenchus avenae* had the highest synteny and shared the highest number of gene families (Wan et al., 2021). A signal peptide and a transmembrane domain, which were distributed in the protein sequences, were also predicted. The signal peptide regulated the integration of *UGT440A1* precursors in the endoplasmic reticulum compartment, whereas the transmembrane domain anchored the protein to the endoplasmic reticulum. Most human UGTs are predominantly expressed in the liver and kidney, which are the major organs involved in detoxification (Meech et al., 2019). In insects, the UGTs are expressed in the fat body, midgut, and other tissues. A study revealed that UGTs are expressed preferentially in the olfactory organ (antenna), indicating that UGTs may be associated with the deactivation of pheromones (Younus et al., 2014). Another study reported the expression of *UGT13* in the hypodermis of *C. elegans* (Hasegawa et al., 2010). In the present study, FISH analysis indicated that *UGT440A1* expresses in all developmental stages of *B. xylophilus*. *UGT440A1* was found to specifically express in the head, intestine, and hypodermis of J2, J3, and J4 females. In J4 males and adults, *UGT440A1* expressed almost in the whole body of *B. xylophilus*, especially in the spicule of adult male nematodes. The results indicated that *UGT440A1* might have multiple functions and a sex-specific role in adult nematodes that have not been identified yet. However, the exact tissue or cells where *UGT440A1* is expressed could not be determined in the present study. Thus, further research is required to determine the protein localization site in *B. xylophilus*.

*UGT440A1* plays diverse roles in motility, feeding, and reproduction of *B. xylophilus. UGT440A1* knockdown was found to impair the motility of the nematodes in the present study. Once *B. xylophilus* have invaded the pines, these nematodes migrate and multiply, thereby producing the symptoms of PWD (Futai, 2013). The cell death and early development of disease symptoms in host trees coincide with *B. xylophilus* migration, and avirulent nematodes migrate into the xylem resin canals much slower than virulent nematodes (Ichihara et al., 2000). This finding suggests that the migration ability may play a crucial role in *B. xylophilus* pathogenicity, especially in the early PWD stage. In *C. elegans*, muscle cell aging and death can affect motility, and reduced insulin-like growth factor (IGF) signaling can prevent muscle cell death (Oh and Kim, 2013). Upregulation of detoxification-related genes including UGTs was found in *daf-2* (the single IGF receptor in *C. elegans*) mutant adults (Gems and McElwee, 2005). The aforementioned findings indicated that *UGT440A1* regulates the motility of nematodes possibly through the IGF signaling pathway, and detoxification may be a possible longevity assurance mechanism in the muscle cells of nematodes.

*UGT440A1* knockdown was also found to reduce the feeding ability of *B. xylophilus.* The nematode feeds on healthy plant tissues of the pine cortex in the early PWD stage, whereas in the later stages of PWD, the nematodes mainly feed on fungi in host trees. A recent study demonstrated that UGTs in *B. xylophilus* are upregulated after infection and are expressed predominantly in the intestine (Espada et al., 2016). In *C. elegans*, the intestine is the first line of defense against exogenous compounds, and phase II detoxification enzymes including UGTs play a key role in this process (Crook-McMahon et al., 2014). Additionally, in the silkworm *Bombyx mori*, UGT (*UGT10286*) promotes the glucosylation of quercetin, facilitates the uptake and transport of quercetin, and influences the overall bioavailability of flavonoids (Daimon et al., 2010). Our results also showed that *UGT440A1* could catalyze the combination of two flavonoids, kaempferol and quercetin, with glucose. These results demonstrated that *UGT440A1* may play a crucial role in digestion and detoxification in the intestine of *B. xylophilus*. However, the mechanisms through which *UGT440A1* aids digestion and detoxification remain to be investigated.

The reproduction ability is closely related to aggressiveness and pathogenicity of plant-parasitic nematodes (Wang et al., 2005). Our results indicated a decrease in the reproduction ability after RNAi of *UGT440A1 in B. xylophilus*. UGTs play a role in steroid metabolism, whereas the sex steroids such as estrogens, androgens, and progesterones are the targets of UGT-mediated metabolism (Meech and Mackenzie, 1997; Meech et al., 2019). UGTs catalyze the glucuronidation of sex steroid hormones, which represents a major pathway of inactivation and excretion of these hormones. In humans, UGTs are expressed in the reproductive organs including uterus, ovary, cervix, placenta, testes, and breast (Meech et al., 2019). The crucial role of several UGT genes in reproduction has also been validated in insects. Knockdown of three UGT genes, namely *UGT352A1*, *UGT352B1* and *UGT354A1*, was shown to reduce the fecundity of female *Bemisia tabaci* (Gennadius) (Guo et al., 2020). Moreover, a recent study reported that UGT12 is a positive modulator of reproduction in brown planthopper because UGT12 knockdown resulted in a decrease in vitellogenin synthesis and hormone acid methyltransferase expression (Ge et al., 2019). Consequently, we speculate that *UGT440A1* influences reproduction in *B. xylophilus* by altering the levels of steroid hormones and other biomolecules.

After *B. xylophilus* invade a pine tree, the host pines generate a wide range of nematicidal and nematistatic substances, which can not only defend the invasion but also decrease the reproduction and migration rate of the nematodes (Futai, 2013; Kuwazu, 1998; He et al., 2016). Therefore, *B. xylophilus* must resist or metabolize those substances to successfully invade the host (Lindblom and Dodd, 2009; Mamiya, 2012). Detoxification-related genes, such as cytochrome P450 genes, have been demonstrated to be essential for feeding, reproduction, and pathogenicity of nematodes (Benenati et al., 2009; Ziniel et al., 2015; Qiu et al., 2019). Moreover, UGTs play a crucial role in actual detoxification reactions (phase II of the detoxification process) of nematodes. The present study demonstrated that PWD onset is markedly delayed after *UGT440A1* knockdown, which highlights the significance of the gene in the pathogenic process of the nematode.

This study is the first to characterize UGTs in *B. xylophilus* and investigate the molecular characteristics and biological functions of *UGT440A1* in *B. xylophilus.* Our study revealed the diverse roles of *UGT440A1* in motility, feeding, and reproduction of *B. xylophilus*. These results suggest that *UGT440A1* gene is involved in the pathogenic process of *B. xylophilus* and the information may facilitate a better understanding of the molecular mechanism of PWD.

## Data Availability Statement

The datasets presented in this study can be found in online repositories. The names of the repository/repositories and accession number(s) can be found in the article/Supplementary Material.

## Author Contributions

MW, RL, and GD designed the experiment. MW and LW performed the RNAi experiments. MW and JF performed the gene cloning experiment. MW and QG performed data analysis. MW, RL, and TZ interpreted data and prepared the manuscript. All authors discussed the results and reviewed the manuscript.

## Conflict of Interest

The authors declare that the research was conducted in the absence of any commercial or financial relationships that could be construed as a potential conflict of interest.

## Publisher’s Note

All claims expressed in this article are solely those of the authors and do not necessarily represent those of their affiliated organizations, or those of the publisher, the editors and the reviewers. Any product that may be evaluated in this article, or claim that may be made by its manufacturer, is not guaranteed or endorsed by the publisher.

## Footnotes

1
https://labs.wsu.edu/ugt/

2
http://parasite.wormbase.org/index.html

3
http://www.ncbi.nlm.nih.gov/gorf/gorf.html

4
https://blast.ncbi.nlm.nih.gov/Blast.cgi

5
www.ncbi.nlm.nih.gov/genbank/

6
https://www.ncbi.nlm.nih.gov/Structure/cdd/

7
https://web.expasy.org/protparam/

8
http://www.cbs.dtu.dk/services/SignalP/

9
http://www.cbs.dtu.dk/services/THHMM/

10
https://swissmodel.expasy.org/
