# Supplementary figures and images for: UGT440A1 Is Associated With Motility, Reproduction, and Pathogenicity of the Plant-Parasitic Nematode Bursaphelenchus xylophilus
Source: Front Plant Sci. 2022 May 31;13:862594. doi: 10.3389/fpls.2022.862594 (PMC9194688; doi:10.3389/fpls.2022.862594)

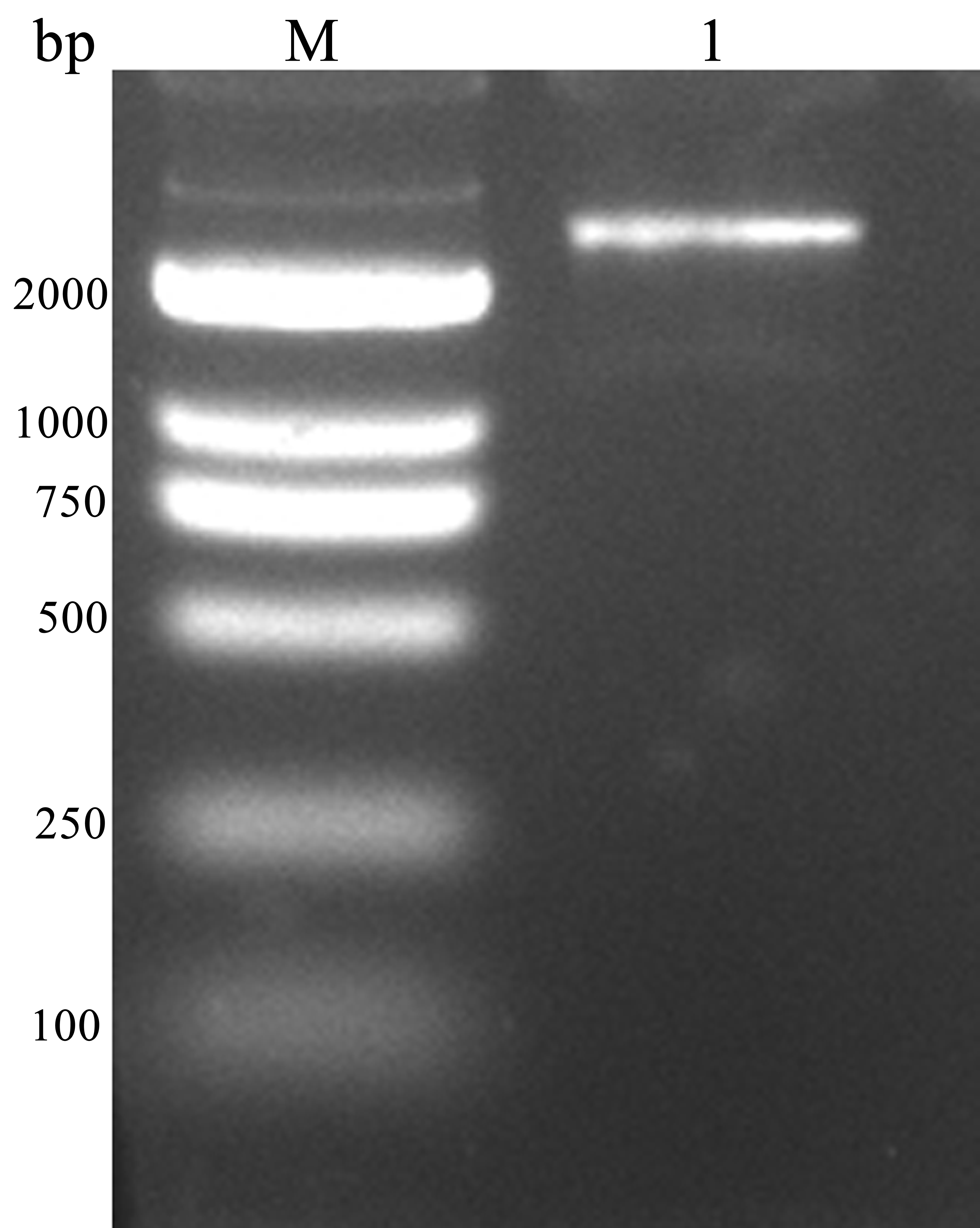

Supplement: Supplementary file 1 [file Image_1.tif]
